# QOI2/356: Trying to Meet the User's Needs in A Web-based Patient Information System

**DOI:** 10.2196/jmir.1.suppl1.e96

**Published:** 1999-09-19

**Authors:** M Lerch, C Reichle, ML Dierks, FW Schwartz

**Affiliations:** ^1^Hannover Medical School, HannoverGermany

**Keywords:** Patient Participation, Evidence-Based Medicine, Quality Control, Public Health, Internet

## Abstract

**Introduction:**

In 1997, a new German Web-based patient information system (www.therapie.net) was developed. As a knowledge base, systematic reviews of the effectiveness of health technologies were used. Widely accepted and additional features were introduced into the system in order to gain a high quality product.

**Methods:**

At the start of the project, text-based patient information was simply converted into HTML format. In the next step, the HON Code principles for medical and health Web sites were implemented. Although the resulting Web pages followed these principles, they were not very user friendly. To improve this deficit all the information available in the system was restructured into main information units. These units determined the basic structure of the information system. Furthermore, the original patient information texts were structured into several divisions and subdivisions, say information levels. This process resulted in a large number of clear text-blocks, each of which showed a branched text navigation bar as well as the content itself.

**Results:**

Merging different solutions for user interfaces and web design, the system was developed on the basis of the frame technique. The implementation of frames facilitates a constant system environment. A system navigation bar is throughout present at the top of the page. It allows the direct and quick access of a specific part of the system -- e.g. introduction, topics, contact, glossary, imprint. Accessing a specific topic, a text navigation bar appears in a left frame. The navigation bars allow the direct selection of the needed information and give a clear message about the user's location in the information space. The different text blocks and system parts are connected through hyperlinks. To avoid confusion, while the user is following such links, another smaller browser window is opened where appropriate. Thus, for example, if a user comes upon a medical term, which is described in the glossary, he can just click on the word and read its description in the smaller window without leaving the original text.

**Discussion:**

A lot of patient information sites in the World Wide Web seem to show a good quality of content, but are lacking user friendliness, and vice versa. To combine these two features, we introduced the widely accepted quality principles of the HON code and developed some own principles, which we presumed to meet the user's needs. However, the techniques and possibilities of the web are changing and developing rapidly. Consequently, all providers of web-based patient information will have to be in pace with this development to ensure the high quality of their products. An online survey (starting in May 1999) will be used to evaluate the quality of our approach.

